# The Flip Side of the Coin: METTL3 Serves as a Novel Cellular Senescence Accelerator *via* Negative Regulation of ITGA9

**DOI:** 10.14336/AD.2024.1715

**Published:** 2025-03-18

**Authors:** Yuting Li, Linying Huang, Miaochun Fang, Liwen Ye, Haiqing Yang, Weijia Wu, Yuan Yuan, Kun Cao, Hui-ling Zheng, Xuerong Sun, Yun Wu, Xing-Dong Xiong, Xinguang Liu, Shun Xu

**Affiliations:** ^1^Institute of Aging Research, Guangdong Provincial Key Laboratory of Medical Immunology and Molecular Diagnostics, School of Medical Technology, Guangdong Medical University, Dongguan, 523808, China.; ^2^Haizhu District Center for Disease Control and Prevention, Guangzhou, China.

**Keywords:** m6A, METTL3, ITGA9, senescence, aging

## Abstract

N6-Methyladenosine (m6A), a prevalent and dynamically regulated chemical modification, has recently emerged as a crucial post-transcriptional regulator of gene expression, and affected diverse eukaryotic biological processes. However, the role of m6A modification in aging research was still rarely reported. Herein, we uncovered that both the m6A modification level and the expression level of the methyltransferase METTL3 were significantly elevated during the aging process, as observed in the physiological aging mouse model in *vivo*, and the cellular senescence model in *vitro*. Furthermore, the silencing of METTL3 staved off the senescent phenotype of MEF cells, as evidenced by the downregulation of p16, decreased β-galactosidase activity and enhanced cell proliferative capacity, while METTL3 overexpression accelerated cellular senescence. Subsequently, a METTL3 transgenic mouse was generated, which exhibited a more pronounced senescence phenotype and a shortened lifespan. To deepen into the understanding of the molecular mechanisms of m6A and METTL3 in the aging process, high-throughput MeRIP sequencing was performed on young and senescent MEFs, and identified ITGA9 as a critical downstream m6A target, which might be negatively regulated by m6A modification or METTL3 through translation inhibition. And loss- or gain-of-function experiments unveiled that ITGA9 remarkably delayed the senescence of MEF cells. Additionally, the inhibition of ITGA9 reversed the impact of METTL3 silencing on delaying senescence, while ITGA9 overexpression counteracted the effect of ectopic expression of METTL3 on advancing cellular senescence. In aggregate, our data suggested that METTL3 promoted cellular senescence by m6A-dependent translational suppression of ITGA9, which was of great significance to alleviate the organismal aging process and age-related diseases.

## INTRODUCTION

Aging is a spontaneous physiological phenomenon characterized by organismal decline, including degenerative lesion, organ atrophy and functional deterioration, and is tightly associated with the progressive accumulation of senescent cells within tissues [1]. Senescent cells exhibit cell-cycle arrest, typically enlarged and flattened morphology and increased activity of senescence-associated β-galactosidase (SA-β-gal) [2]. Recent years, N6-methyladenosine (m6A) modifications have been established to function as novel regulators of gene expression at the posttranscriptional level and thus have been involved in various physiological and pathological processes. Hence, the pivotal role of m6A modifications in modulating the aging process and aging-related genes has gained increasing attention since the discovery of the first RNA demethylase FTO [3, 4].

As a highly conserved, dynamic and reversible chemical modification of RNA, m6A methylation is the most prevalent modification within eukaryotic messenger RNA (mRNA) and long non-coding RNAs (lncRNAs), which is enriched in long internal exons, proximal to stop codons and within 3′UTRs, and usually occurs within the consensus motif RRACH ( [G/A/U] [G > A] m6 AC [U > A > C]) [5]. The m6A methylation is catalyzed by the core methyltransferase complex consisted of METTL3, METTL14, WTAP, etc. (termed “writers”), and is removed by the demethylases FTO and ALKBH5 (termed “erasers”). Additionally, m6A binding proteins (termed “Readers”), including YTHDF1/2/3, YTHDC1 and HNRNP, specifically recognize and bind to the m6A-methylated transcripts, thereby determine their fates by affecting the splicing, transportation, stability and translation [6]. Recent evidence has increasingly revealed the close association of m6A modification with various biological processes, indicating a potential role for m6A methylation in the aging process.

METTL3 (Methyltransferase 3) serves as the principal component of the methyltransferase complex, which possesses the catalytic activity in N6-methyladenosine (m6A) modification. A growing body of evidence has unveiled that the aberrant expression of METTL3 disrupted multiple physiological processes, including DNA damage repair [7], immune response [8], spermatogoniogenesis [9], hematopoiesis [10], circadian rhythms [11] and cell cycles [12], by affecting gene expression through altering the m6A modification level. Recent studies have indicated a potential role for m6A modification in cellular senescence [13, 14]. Nonetheless, the relationship between m6A methylation and cellular senescence, as well as the underlying mechanisms, remains poorly defined, and appears to be inconsistent or heterogenous through previous studies, which attracted our interests to explore this issue in greater depth.

In this study, we uncovered that both the m6A modification level and METTL3 expression were dramatically enhanced during the aging process, which substantially accelerated cellular senescence. Furthermore, high-throughput MeRIP-seq was performed to identify novel aging-related genes regulated by m6A methylation and thus shed light on the effect and underlying molecular mechanism of METTL3 on cellular senescence.

## MATERIALS AND METHODS

### MEFs preparation, Cell culture and Mouse tissues

Mouse embryonic fibroblasts (MEFs) were derived from C57BL/6 mice. Embryos were isolated on embryonic day 13.5 (E13.5). Embryo was minced and digested with trypsin at 37°C for 15 minutes. Following a two-day culture period, the MEFs were cryopreserved in liquid nitrogen and designated as passage 0 (P0). NIH/3T3 fibroblasts were procured from the American Type Culture Collection (ATCC). Above cell types were cultured in Dulbecco's Modified Eagle Medium (DMEM) supplemented with 10% fetal bovine serum (FBS).

By serial passaging of MEFs, a replicative senescence model was established, with passage 3 (P3) representing young cells and passage 9 (P9) representing senescent cells. To induce premature senescence in NIH/3T3 fibroblasts, oxidative stress was applied using 500 μM hydrogen peroxide (H_2_O_2_) for 6 hours, thereby establishing an oxidative stress-induced premature senescence model.

The C57BL/6 mouse strain is a widely utilized inbred laboratory model with a well-characterized genetic background. As a prototypical "black 6" strain, C57BL/6 mice exhibit a homozygous recessive genotype for coat color (a/a, B/B, C/C) and are characterized by robust metabolic profiles, age-related hearing loss, and susceptibility to diet-induced obesity. Due to their genetic stability, reproducible phenotypes, and compatibility with genetic engineering technologies (e.g., CRISPR/Cas9), C57BL/6 mice are considered a gold standard for studies in immunology, neuroscience, metabolic disorders, and aging research.

Mouse tissues were collected from male C57BL/6 mice aged 2 months and 20 months. The tissues were individually disrupted using ultrasonic technology in lysis buffers prior to RNA extraction, or protein analysis. The research complied with the guidelines and ethical standards of the Chinese Council on Animal Care and received approval from the Medicine Ethics Committee of Guangdong Medical University.

### Antibodies

M6A antibody (202003, Synaptic Systems, Germany), GAPDH Monoclonal antibody (60004-1-Ig, Proteintech, China), METTL3 Monoclonal antibody (ab195352, Abcam, UK), FTO Polyclonal antibody (27226-1-AP, Proteintech, China), METTL14 Polyclonal antibody (AP22363a, Abgent, USA), ALKBH5 Polyclonal antibody (16837-1-AP, Proteintech, China), WTAP Monoclonal antibody (60188-1-Ig, Proteintech, China), p16 INK4A antibody (F-12) (sc-1661, Santa Cruz, USA), ITGA9 Polyclonal antibody (AF3827, R&D, USA), BrdU Monoclonal antibody (66241-1-Ig, Proteintech, China), Goat Anti-Mouse antibody (Alexa Fluor® 488) (SA00013-1, Proteintech, China), Goat anti-rabbit IgG (H+L) antibody (A0208, Beyotime, China), Goat anti-mouse IgG (H+L) antibody (A0216, Beyotime, China), Donkey anti-goat IgG (H+L) antibody (A0181, Beyotime, China).

### RNA extraction, Reverse transcription and qPCR

Total RNA was extracted from the cells using Trizol Reagent (Thermo Fisher Scientific, USA), and RNA purity and concentration were assessed by measuring the A260/A280 nm ratio. RNA integrity was evaluated using agarose gel electrophoresis.

The PrimeScriptTM RT reagent Kit (Takara, Japan) was employed to convert 1μg of RNA into cDNA. SYBR Select Master Mix (Takara, Japan) was utilized to performing qRT-PCR on a LightCycler 96 (Roche, Switzerland), with the beta-actin gene serving as an internal control. The primers used are listed in Supplementary Table 1.

### RNA oligoribonucleotides and cell transfections

The siRNAs were designated as siMETTL3-1/2/3 and siITGA9-1/2/3. These RNA oligoribonucleotides were synthesized by Genepharma (China) and listed in Supplementary Table 2. For siRNA transfections, Lipofectamine RNAiMAX (Thermo Fisher Scientific, USA) was utilized. Briefly, a mixture of siRNA (50nM) and Lipofectamine RNAiMAX (2μl) was incubated with cells for 6h.

### Plasmid construction, Lentivirus packaging and infection

To achieve overexpression of METTL3 and ITGA9 in MEF cells, lentiviral vectors were constructed and packaged. The full-length coding sequence of METTL3 was cloned and inserted into the GV492 vector (Ubi-MCS-3FLAG-CBh-gcGFP-IRES-puromycin). Similarly, the full-length coding sequence of ITGA9 was cloned and inserted into the GV367 vector (Ubi-MCS-SV40-EGFP-IRES-puromycin). The processes of plasmid construction and lentivirus packaging were carried out by Shanghai Jikai Company (China). Lentivirus infection was conducted at a multiplicity of infection (MOI) of 20, utilizing the HiTransG P reagent provided by Shanghai Jikai Company according to the manufacturer’s instruction.

### M6A dot blotting assay

Total RNA was extracted using the Trizol reagent (Thermo Fisher Scientific, USA), and mRNA was subsequently isolated from the total RNA using the PolyATtract^R^ mRNA Isolation System IV (Promega Z5310, USA) according to the manufacturer’s instruction. The mRNAs were denatured by heating at 65°C for 10 minutes and then immediately placed on ice for 10 minutes to prevent renaturation. The amount of 100ng-1000ng of mRNA was loaded onto the Nylon Transfer Membrane (Amersham, GE Healthcare, USA). Subsequently, the membrane was subjected to UV cross-linking at 1200 KJ, followed by blocking with 5% skimmed milk powder for 1 hour at room temperature and then incubated with an m6A antibody (dilution 1:500, Synaptic Systems, Germany) overnight at 4°C, and with an HRP-conjugated goat anti-rabbit IgG (dilution 1:2500) for 1 hour at room temperature. The results of the dot blotting were detected using the Immobilon Western HRP Substrate (Millipore, Germany). Methylene blue (MB)staining was used as a loading control.

### Senescence-associated-galactosidase (SA-Gal) staining

SA-β-gal staining was performed utilizing the X-gal staining kit (Sigma-Aldrich, Germany). Cells were washed with PBS, fixed in a solution containing 2% formaldehyde and 0.2% glutaraldehyde in PBS, and then incubated with a fresh β-galactosidase staining solution at pH 5.8 at 37°C at least 12 hours. Finally, cells were observed and photographed under an inverted bright field microscope (Nikon, Japan) at 100× magnification. The percentage of SA-β-gal-stained cells was determined by counting the number of blue-stained cells in a sample containing more than 800 cells.

### Cell proliferation assay

BrdU incorporation was performed to evaluate cell proliferation. Cells were incubated with 40μM BrdU for 1 hour in an incubator. Subsequently, cells were fixed with 4% formaldehyde for 15minutes and digested with trypsin solution at 37°C for 7minutes. Subsequently, the cells were treated with 4M HCl to denature the DNA. Then the cells were blocked with an incubation buffer containing 3% BSA and 0.1% Tween-20, and this process was carried out overnight at 4°C. Following this, the cells were incubated with an anti-BrdU primary antibody (1B10E12, Proteintech, China) for 1 hour at 37°C, followed by a 1-hour incubation with a fluorescent secondary antibody (SA00013-1, Proteintech, China). Finally, DAPI (Sigma-Aldrich, Germany) was used to counterstain the nuclei, and the cells were promptly visualized using a fluorescence microscope (Olympus, Japan).

To eliminate non-specific binding of the primary antibody, we used an isotype-matched control mouse antibody at the same concentration as the experimental antibody. This control confirmed that no signal was observed in the target area, indicating that the primary antibody is specific to its antigen. Samples were incubated separately with the secondary antibody alone (without the primary antibody), and little/no background staining was observed, confirming that the secondary antibody does not cause false-positive signals. Furthermore, Samples were not treated with either the primary or secondary antibodies, serving as a control to ensure that the observed staining pattern was not due to endogenous autofluorescence or non-specific interactions with the detection system.

### Animal studies

C57BL/6J mice were procured from the Shanghai Model Organisms Center, Inc. (Shanghai, China). The Rosa26^CAG-METTL3^ knock-in mice were generated at the same facility. The CAG-METTL3-WPRE-polyA cassette was inserted into the Gt (ROSA)26Sor (Rosa26) locus by the CRISPR-Cas9 methodology. Genotyping of all mouse strains was conducted via PCR with the following primers: wild-type allele (994 bp), P1: 5'-TCAGATT CTTTTATAGGGGACACA-3', P2: 5'-TAAAGGCCAC TCAATGCTCACTAA-3', knock-in allele (746 bp), P3: 5'-AGGGCCGAAGGGACGTAGCAGAAG-3', P4: 5'-A GGGCCTGGACTGCGATGTGATTG-3'. The breeding and maintenance of the mice adhered strictly to protocols approved by the Institutional Animal Care and Use Committee of Guangdong Medical University (Approval No. 202104A075). The mice were housed under a 12-hour light/dark cycle and specific-pathogen-free conditions. The temperature within the animal facility was controlled between 20 and 24 °C, with humidity levels maintained between 45% and 65%.

### Treadmill Measurement

Prior to testing, mice underwent training on an animal experimental treadmill (Beijing Zhongshi Dichuang Technology Development Co.) with a fixed 3-degree incline and a constant speed of 10 m/min for 5 minutes daily over three consecutive days. On the day of exercise testing, the animals ran on the treadmill at 10 m/min for 5 minutes, followed by 15 m/min for 5 minutes, and then at a maximum speed of 20 m/min until exhaustion was reached. Exhaustion was defined as the animal's inability to continue running on the treadmill for a duration of 10 seconds, despite external mechanical stimulation. Measurements were taken for running time and maximum speed attained, while running distance was calculated.

### Forelimb grip strength test

A mouse grip strength meter (KW-ZL-2, Calvin Biotechnology Co., Nanjing, China) was positioned horizontally, equipped with a rigid grid attached to the force transducer. The mouse was permitted to grasp the grid with its forepaws and then pulled away from the grid by its tail until its paws released the grid, at which point the force exerted was recorded. This procedure was repeated three times, with a five-minute interval between each test.

### Micro-CT imaging and analysis

To determine potential differences in spinal curvature, adiposity, and bone development among distinct groups of mice, the mice was subjected to undergo scanning and analysis via a micro-CT imaging system (microCT Quantum GX2, Revvity). The mice were fasted for six hours prior to scanning to reduce the impact of residual food on the experimental results, although water was provided. Before scanning, anesthesia was induced using 2% isoflurane in a pre-anesthetic chamber. Following the administration of anesthesia, the mice were positioned horizontally on the cuvette with their ventral side facing downward, while the apparatus was continuously supplied with 2% isoflurane. The mice were scanned under the following conditions: voltage at 50 kV, current at 100 μA, and a field of view (FOV) of 36 mm. The scanning duration was set to 2 minutes, and the X-ray filter was composed of five segments. All values set to 1.0.

After scanning, the computed tomography (CT) images were analyzed using Analysis 14.0 software. Firstly, the raw data were processed using the “Spatial Filter” under “Process”, and all the samples were filtered under the following conditions: “Median, Kernel Size, X:3 mm; Y:3 mm; Z:3 mm”. Subsequently, the “Threshold Volume” was used. All samples were filtered by “Median, Kernel Size, X:3 mm; Y:3 mm; Z:3 mm”. All samples were subjected to the median filter with the specified kernel size, followed by the extraction of the mouse skeleton through threshold value adjustments using "Threshold Volume". The skeleton was then analyzed using "Edge Volume". To enhance the edge definition and facilitate improved segmentation of adipose tissue, "Edge Strength" was applied, and “Region Grow” was used to extract whole-body fat for the calculation of whole-body bone mineral density (BMD). Ar/Tt. Ar (Cortical Area Fraction, %), Ct. Th (Average Cortical Thickness, mm), and Ct. Ar (Cortical Bone Area, mm^2^) were calculated by selecting the area of interest in the femur. Ar (Cortical Bone Area, mm^2^). Spinal Curvature (Degrees) was calculated by selecting the spinal region of interest. Finally, the "Display" function was used to save the image.

### MeRIP-sequencing

High throughput m6A sequencing were performed under the support of Kangchen Biotech Company (Shanghai, China). Briefly, total RNA was extracted from MEF cells at passages P3 and P9. The quality of RNA was assesed, and then randomly fragmented into approximately 150 nucleotides segments by RNA fragmentation reagents. The fragmented RNA was then incubated with an m6A-specific primary antibody (Synaptic Systems, Germany) at 4°C for 2 hours to facilitate immunoprecipitation. Following, the eluted RNA was precipitated by 75% ethanol and treated with RNasin (Ambion, USA) according to the manufacturer’s instructions. The TruSeq Stranded mRNA Sample Pre-Kit (Illumina, USA) was utilized to prepare the library from immunoprecipitated RNA and input RNA. Sequencing was performed on an Illumina HiSeq machine with paired-end sequencing.

### MeRIP-qPCR

Total RNA was extracted using Trizol Reagent, and mRNA was isolated from the total RNA using the PolyATrct^R^ mRNA Isolation System IV. MeRIP was performed using the Magna MeRIP™ m6A Kit (Millipore, Germany). During the MeRIP procedure, RNA was chemically fragmented into approximately 100 nucleotides segments by RNA Fragmentation Buffer, followed by immunoprecipitation with a monoclonal antibody against m6A provided in the kit. The RNase MiniElute Kit (Qiagen, Germany) was used to extract RNA from the solution. After immunoprecipitation, the isolated RNA was analyzed with qRT-PCR.

### Western blotting

Cell protein lysates were loaded on a 12% SDS-polyacrylamide gel, followed by electrophoretical transfer onto an Immobilon-PSQ PVDF membrane (0.2μm pore size) (Millipore, Germany). Then the membrane was blocked with 5% skimmed milk powder, and incubated with the primary antibody overnight at 4°C. Next, the membrane was incubated with an HRP-conjugated secondary antibody. Finally, the results were detected with the Immobilon Western HRP Substrate (Millipore, Germany), with the GAPDH antibody employed for normalization of protein load.

### Nucleo-cytoplasmic separation assay

Nucleo-cytoplasmic separation assay was used to determine the subcellular localization of ITGA9 with Cytoplasmic & Nuclear RNA Purification Kit (Thermo Fisher Scientific, USA). Cells were digested and collected, and then cell membranes were lysed by Cell Fraction Buffer. After centrifugation, the supernatant was collected as the cytoplasmic fraction, while the precipitated nuclears were lysed by Cell Disruption Buffer to obtain the nuclear fraction. Finally, RNA was extracted using Trizol Reagent for subsequent qRT-PCR analysis.

### Statistical analysis

The data were presented in three formats: standardized probabilities (e.g., gene and protein expression levels), raw data (e.g., the fat volume, bone density, and other related measurements in mice), and raw statistical values (e.g., BrdU incorporation assays and SA-β-gal staining). For comparisons of standardized probabilities, we first employ the Shapiro-Wilk test to assess whether the normalized ratios conform to a normal distribution, followed by a one-sample t-test. For the raw data measured in mice, we initially applied the Shapiro-Wilk test to evaluate the normality of the data and subsequently performed an Unpaired *t*-test. For raw statistical values, we similarly use the Shapiro-Wilk test to evaluate whether the data adhere to a normal distribution and subsequently perform a paired *t*-test.

### Accession number

METTL3 coding sequence (NCBI Reference Sequence: NM_019721.2) was obtained from NCBI (www.ncbi.nlm.nih.gov/). The coding sequences of ITGA9 (NCBI Reference Sequence: NM_001113514.1) were also obtained from NCBI.

## RESULTS

### The m6A modifications within total mRNA were significantly enhanced during aging process

**Figure 1.**
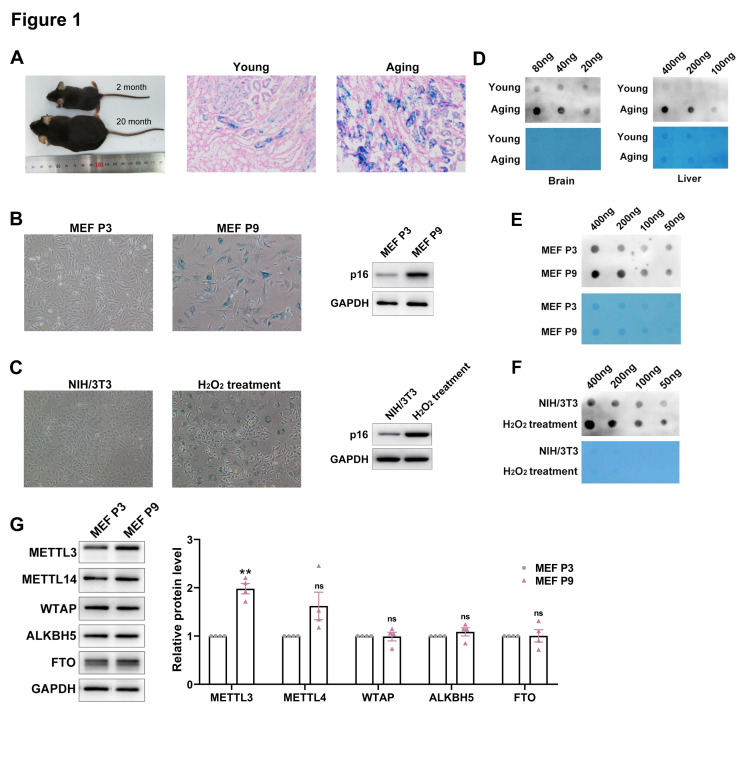
**M6A modifications within total mRNA were significantly enhanced during the aging process**. (**A**) Left: representative photographs of 2-month and 20-month-old male C57BL/6 mouse; Right: representative photographs of SA-β-gal staining in kidney tissues from young and old C57-BL/6 mice. (**B**) Left: representative photographs of SA-β-gal staining of P3 and P9 MEF cells; Right: the p16 expression levels in P3 and P9 MEF cells. (**C**) Left: representative photographs of SA-β-gal staining of H_2_O_2_-induced premature senescence of NIH/3T3 cells; Right: the p16 expression levels in H_2_O_2_ treated NIH/3T3 cells. (**D**) Poly(A)+ RNA was extracted from the brain and liver tissue of young and old C57-BL/6 mice and subjected to RNA dot-blot analysis with an antibody recognizing m6A. Methylene blue staining served as the loading control. (**E**) Poly(A)+ RNA was extracted from the P3 and P9 MEF cells and subjected to RNA dot-blot analysis with an antibody recognizing m6A. Methylene blue staining served as the loading control. (**F**) Poly(A)+ RNA was extracted from the P3 and P9 MEF cells and subjected to RNA dot-blot analysis with an antibody recognizing m6A. Methylene blue staining served as the loading control. (**G**) Expression levels of m6A modification associated proteins were measured in P3 and P9 MEF cells, and the quantitative results were shown on the right. Mean (±SEM), n=4, passed normality test: Shapiro-Wilk test; One sample *t* test (MEF P9 vs 1, METTL3:*p*=0.0027, *t*= 9.176).

To evaluate the m6A methylation levels during the aging process, we established a physiological aging mouse model using 20-month-old male C57BL/6 mice as the aging group, in comparison to the 2-month-old male C57BL/6 mice as the young group (Fig. 1A). Additionally, Mouse Embryonic Fibroblasts (MEFs) were utilized to establish a replicative senescence cell model, which exhibited typical senescence- associated phenotypes after serial passage (passage 3 as young MEFs and passage 9 as senescent MEFs) (Fig. 1B). Moreover, an oxidative stress-triggered premature senescence model of NIH/3T3 cells was generated through H_2_O_2_ treatment (Fig. 1C).

Subsequently, an m6A dot blot assay was performed to assess the m6A level of RNA epitranscriptome (mRNA) in the established aging models. The results showed that m6A level of total mRNAs was dramatically enhanced in both aging mice and senescent MEFs (Fig. 1D and 1E). Additionally, an up-regulation of m6A level was observed in H_2_O_2_-induced premature senescent NIH/3T3 cells as well (Fig. 1F). Furthermore, we evaluated the expression levels of m6A-related genes during the aging process. The data revealed that the METTL3 protein level was significantly increased in senescent cells and tissues, whereas other proteins showed no distinctive difference (Fig 1G). These results indicated that m6A methylation and METTL3 might exert crucial roles in the regulation of senescence.

**Figure 2.**
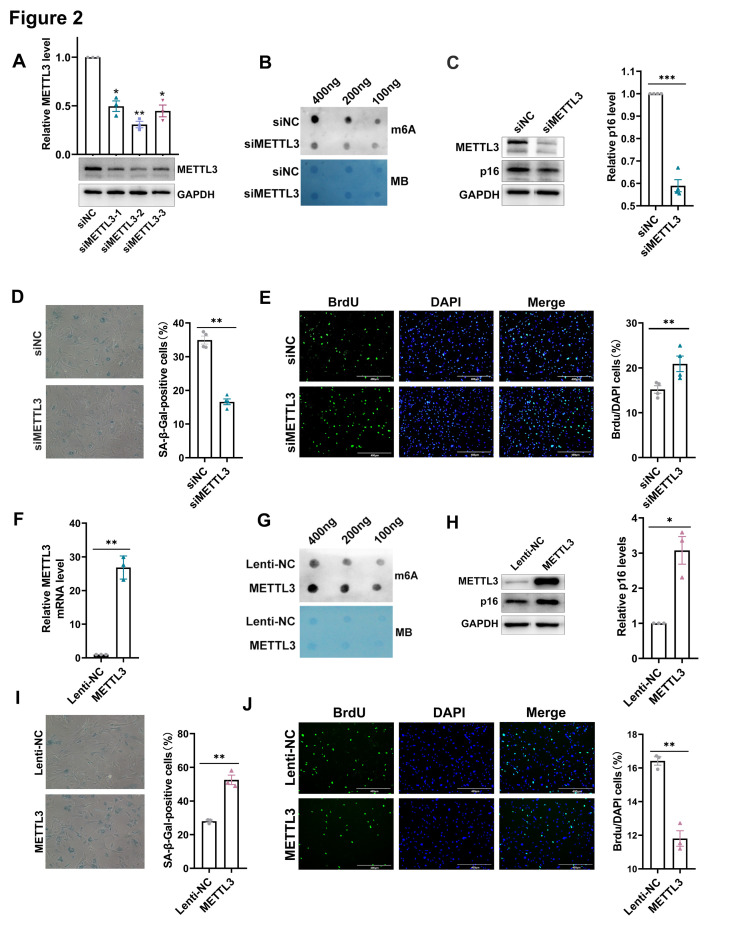
**METTL3 accelerated replicative senescence in MEFs**. (**A**) The protein levels in MEFs transfected with siNC and siMETTL3-1/2/3. Mean (±SEM), n=3, passed normality test: Shapiro-Wilk test; One sample *t* test (siMETTL3-1 vs 1: *p*=0.0113, *t*=9.328; siMETTL3-2VS 1:*p*=0.0022, *t*=21.05; siMETTL3-3 vs 1:*p*=0.0117, *t*= 9.155). (**B**) Poly(A)+ RNA was extracted from MEF cells transfected with siNC and siMETTL3 and subjected to RNA dot-blot analysis with an antibody recognizing m6A. Methylene blue staining served as the loading control. (**C**) Western blot assay of p16 expression in MEFs transfected with siNC and siMETTL3. The quantitative results were shown on the right. Mean (±SEM), n=4, passed normality test: Shapiro-Wilk test; One sample *t* test (siMETTL3 vs 1, *p*=0.0006, *t*=15.36). (**D**) Representative photographs of SA-β-gal staining of MEF cells transfected with siNC and siMETTL3 (×100). The quantitative results of SA-β-gal staining were shown on the right. Mean (±SEM), n=4, passed normality test: Shapiro-Wilk test; Paired *t* test (siMETTL3 vs siNC, *p*=0.0025, *t*=9.407). (**E**) Representative photographs of cells stained with DAPI (blue fluorescence) and BrdU (green fluorescence) in siNC and siMETTL3 transfected MEF cells (scale bar: 400μm). The quantitative results of the ratio (BrdU stained cells / DAPI stained cells) were shown on the right. Mean (±SEM), n=4, passed normality test: Shapiro-Wilk test; Paired *t* test (siMETTL3 vs siNC, *p*=0.009, *t*=6.065). (**F**) The mRNA levels in MEFs infected with Lenti-NC and METTL3 lentivirus. Mean (±SEM), n=3, passed normality test: Shapiro-Wilk test; One sample *t* test (METTL3VS 1, *p*=0.0058, *t*=13.03). (**G**) Poly(A)+ RNA was extracted from MEF cells infected with Lenti-NC and METTL3 lentivirus and subjected to RNA dot-blot analysis with an antibody recognizing m6A. Methylene blue staining served as the loading control. (**H**) Western blot assay of METTL3 and p16 expression in MEFs infected with METTL3 lentivirus. The quantitative results were shown on the right. Mean (±SEM), n=3, passed normality test: Shapiro-Wilk test; One sample *t* test (METTL3 vs 1, *p*=0.0342, *t*=5.271). (**I**) Representative photographs of SA-β-gal staining of MEF cells infected with Lenti-NC and METTL3 lentivirus (×100). The quantitative results of SA-β-gal staining were shown on the right. Mean (±SEM), n=3, passed normality test: Shapiro-Wilk test; Paired *t* test (METTL3 vs Lenti-NC, *p*=0.0088, *t*=10.59). (**J**) Representative photographs of cells stained with DAPI (blue fluorescence) and BrdU (green fluorescence) in Lenti-NC and METTL3 lentivirus infected MEF cells (scale bar: 400μm). The quantitative results of the ratio (BrdU stained cells / DAPI stained cells) were shown on the right. Mean (±SEM), n=3, passed normality test: Shapiro-Wilk test; Paired *t* test (METTL3 vs Lenti-NC, *p*=0.0065, *t*=12.35).

### METTL3 accelerated cellular senescence

To investigate the impact of METTL3 on cellular senescence, siRNAs specifically targeting METTL3 (designated as siMETTL3-1/2/3) were designed and synthesized. The inhibition of METTL3 in MEF cells was confirmed. Among the siRNAs, siMETTL3-2 exhibited the highest suppression efficiency in MEFs, thus was employed in subsequent experiments, and designated as siMETTL3 (Fig. 2A). Western blot and m6A dot blot assay showed that siMETTL3 significantly reduced m6A level in MEFs. Senescence marker p16 and SA-β-galactosidase staining were used to evaluate cellular senescence, and BrdU incorporation was utilized to determine the proliferative capacity. The results revealed that silencing of METTL3 delayed replicative senescence in MEFs, as evidenced by the downregulation of p16, decreased activity of senescence-associated β-galactosidase and enhanced cell proliferative capacity (Fig. 2C, 2D and 2E).

Moreover, a lentivirus containing the coding sequence of METTL3 was prepared (designated as METTL3), and its overexpression of METTL3 was confirmed by qPCR and western blot (Fig.2F and 2H). The m6A dot blotting assay showed that the ectopic expression of METTL3 significantly enhanced the global m6A level in MEFs as expected (Fig. 2G). The impact of METTL3 overexpression on cellular senescence was evaluated in terms of β-galactosidase activity, p16 expression level and cell proliferative capacity. The results unveiled that the ectopic expression of METTL3 endowed MEFs with elevated p16 level, enhanced β-galactosidase activity and diminished proliferative capacity (Fig. 2H, 2I and 2J). Collectively, these results suggested that METTL3 accelerated replicative senescence in MEFs, underscoring the critical role of m6A modification in the regulation of cellular senescence.

To further verify the impact of METTL3 in cellular senescence, NIH/3T3 cells were transfected with either siMETTL3 or siNC (Supplementary Fig.1A), followed by treatment with hydrogen peroxide (H_2_O_2)_. Expectedly, loss of METTL3 conferred NIH/3T3 cells with reduced p16 level, decreased β-galactosidase activity, and enhanced proliferation (Supplementary Fig.1B, 1C and 1D), which suggested that silencing of METTL3 counteracted the premature senescence induced by H_2_O_2_. Additionally, NIH/3T3 cells were infected with the METTL3 overexpressed lentivirus or Lenti-NC. The results revealed that METTL3 overexpression significantly elevated the p16 level and β-galactosidase activity, and repressed cell proliferation (Supplementary Fig.1F, 1G and 1H). Hence, we concluded that METTL3 accelerated cellular senescence in *vitro*, which indicated the crucial involvement of m6A modification on senescence.

### METTL3 transgenic mouse exhibited shortened lifespan

**Figure 3.**
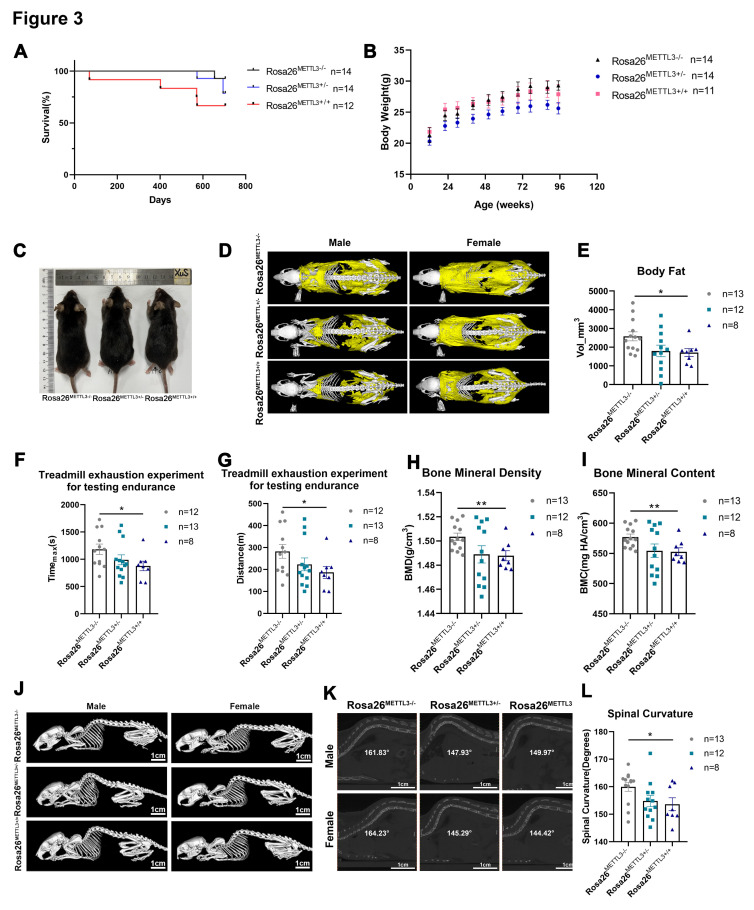
**METTL3 transgenic mouse exhibited shortened lifespan**. (**A**) Kaplan-Meier survival curve analysis for wild-type (Rosa26^METTL3-/-^), heterozygous (Rosa26^METTL3+/-^) and homozygous (Rosa26^METTL3+/+^) mice. n=14,14,12 (B) The body weight of Rosa26^METTL3-/-^, Rosa26^METTL3+/-^, and Rosa26^METTL3+/+^ groups. n=14,14,12. (**C**) Representative photographs of hair and body characteristics in Rosa26^METTL3-/-^, Rosa26^METTL3+/-^, and Rosa26^METTL3+/+^ groups. (**D**) The representative image of body fat revealed by microcomputed tomography (micro-CT) in mice (23 months old) from Rosa26^METTL3-/-^, Rosa26^METTL3+/-^, and Rosa26^METTL3+/+^ groups. The body fat in the mice was colored yellow. (**E**) The body fat volume in Rosa26^METTL3-/-^, Rosa26^METTL3+/-^, and Rosa26^METTL3+/+^ groups. Mean (±SEM), n=13,12,8, passed normality test: Shapiro-Wilk test; Unpaired *t* test (Rosa26^METTL3+/+^ groups vs Rosa26^METTL3-/-^, *p*=0.0238, *t*=2.457). (F, G) Analysis of treadmill exhaustion running distance (F) and treadmill exhaustive running time (G) in Rosa26^METTL3-/-^, Rosa26^METTL3+/-^, and Rosa26^METTL3+/+^ groups. Mean (±SEM), n=12,13,8, passed normality test: Shapiro-Wilk test; Unpaired *t* test((F): Rosa26^METTL3+/+^ groups vs Rosa26^METTL3-/-^, *p*=0.0394, *t*=2.221; (G): Rosa26^METTL3+/+^ groups vs Rosa26^METTL3-/-^, *p*=0.443, *t*=2.162). (H, I) Analysis of BMD (bone mineral density) and BMC (bone mineral content) using micro-CT in Rosa26^METTL3-/-^, Rosa26^METTL3+/-^, and Rosa26^METTL3+/+^ groups. Mean (±SEM), n=13,12,8, passed normality test: Shapiro-Wilk test; Unpaired *t* test ((H): Rosa26^METTL3+/+^ groups vs Rosa26^METTL3-/-^, *p*=0.0065, *t*=3.056; (I): Rosa26^METTL3+/+^ groups vs Rosa26^METTL3-/-^, *p*=0.0065, *t*=3.056). (J, K) X-ray presentation of kyphoscoliosis in Rosa26^METTL3-/-^, Rosa26^METTL3+/-^, and Rosa26^METTL3+/+^ groups. (**L**) Quantification of kyphotic tortuosity. Mean (±SEM), n=13,12,8, passed normality test: Shapiro-Wilk test; Unpaired *t* test (Rosa26^METTL3+/+^ groups vs Rosa26^METTL3-/-^, *p*=0.0347, *t*=2.275).

To elucidate the role of m6A modification in the aging process in *vivo*, we generated METTL3 transgenic mice by knocking CAG-METTL3-WPRE-polyA cassette into the Rosa26 locus utilizing the CRISPR/CAS9 technology, which were classified as wild-type (Rosa26^METTL3-/-^), heterozygous (Rosa26^METTL3+/-^) or homozygous (Rosa26^METTL3+/+^). The mice were maintained in a specific pathogen-free (SPF) environment until natural death, and their lifespans were recorded. Analysis of survival curves showed a drastically reduced lifespan in the Rosa26^METTL3+/+^ mice (n=12) compared to the Rosa26^METTL3-/-^ mice (n=13). Nevertheless, the Rosa26^METTL3+/-^ group (n=13) showed no significant differences in lifespan compared to the Rosa26^METTL3-/-^ group (Fig. 3A).

As shown in Fig.3C, the hair of mice in the Rosa26^METTL3+/+^ group appeared slightly sparse, dull, and lacking in luster compared with the control Rosa26^METTL3-/-^ group (Fig. 3C). Otherwise, the overall appearance and body weight of the Rosa26^METTL3+/+^ mice were indistinguishable from that of the control mice (Fig. 3B and 3C). Aging was usually accompanied by an increase in visceral fat deposition and a reduction in subcutaneous fat. Thus, we analyzed fat distribution using micro-computed tomography (micro-CT), which revealed that Rosa26^METTL3+/+^ mice exhibited a reduction in overall fat and a strikingly diminished hypodermal fat (Fig. 3D and 3E). To assess muscle strength, mice were subjected to a treadmill exhaustion test, which showed that Rosa26^METTL3+/+^ mice exhibited significantly shorter running times and distances, indicating decreased exercise endurance compared to Rosa26METTL3^-/-^ controls (Fig. 3F and 3G). Age-related bone loss leads to a reduction in bone mineral density (BMD) and the development of osteoporosis. The data of Micro-CT analysis revealed that the BMD values of the Rosa26^METTL3+/+^ mice were lower than those of the control Rosa26^METTL3-/-^ group (Fig. 3H and 3I). The skeletal alterations with age generally manifested as kyphoscoliosis. The kyphotic index in Rosa26^METTL3+/+^ mice was higher than that in the control Rosa26^METTL3-/-^ group (Fig. 3J,3K and 3L). Collectively, these results suggested that Rosa26^METTL3+/+^ mice exhbited more pronounced senescence features.

### Transcriptome-wide profiling of m6A modifications in MEFs replicative senescence

To identify the downstream m6A targets involved in the regulation of cellular senescence, methylated RNA immunoprecipitation sequencing (MeRIP-seq) was performed to describe the transcriptome-wide m6A landscape in P3 MEFs and P9 MEFs. The analysis identified 840 up-regulated and 203 down-regulated m6A peaks in the senescent MEFs (P9) compared to young MEFs (P3), which was consistent with the results presented by m6A dot blotting assay (Supplementary Fig. 2A). Notably, among these significantly different peaks, various senescence-associated genes, such as SIRT1 and IRS2, exhibited higher m6A levels in their transcripts in senescent MEFs compared to that in young MEFs. What’s more, KEGG pathway analysis unveiled that the differentially m6A peaks were enriched in multiple aging-associated pathways, including FOXO and PI3K-AKT signaling pathways (Supplementary Fig.2B). These results indicated that METTL3 might modulate the expression of these senescence-related genes in a m6A modification-dependent manner, thereby promoting cellular senescence.

### ITGA9 was negative correlation with METTL3

**Figure 4.**
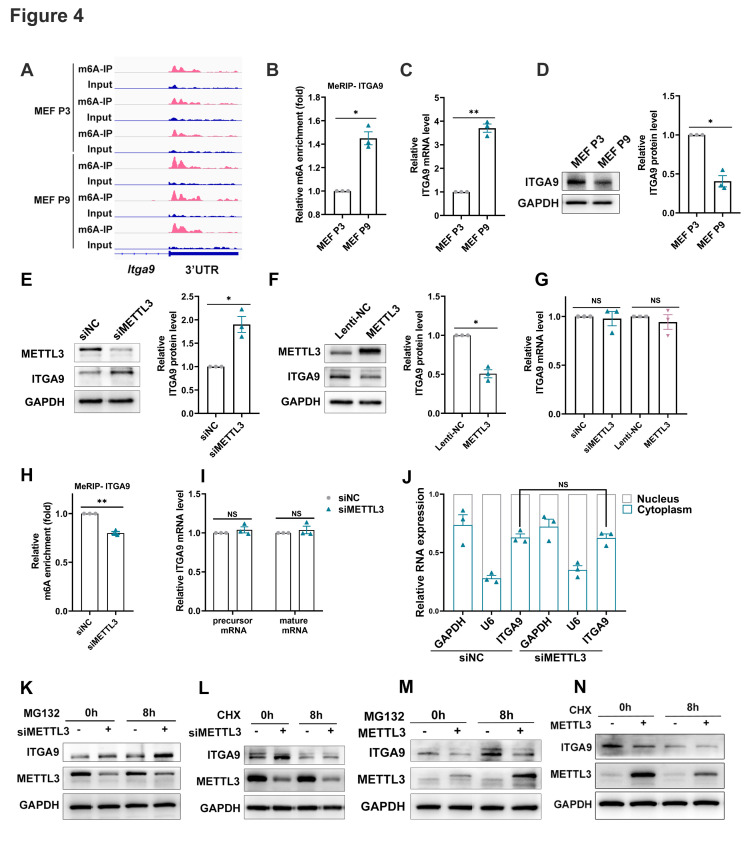
**ITGA9 was negative correlation with METTL3**. (**A**) M6A peaks in the ITGA9 3’UTR transcript based on MeRIP-seq data in P3 and P9 MEF cells. (**B**) Detection of m6A modification within ITGA9 mRNA in P3 MEFs and P9 MEFs using MeRIP-qPCR. Mean (±SEM), n=3, passed the normality test: Shapiro-Wilk test; One sample *t* test (MEF P9 vs 1:*p*=0.0147, *t*=8.145). (**C**) The mRNA levels of ITGA9 in P3 MEFs and P9 MEFs. Mean (±SEM), n=3, passed normality test: Shapiro-Wilk test; One sample *t* test (MEF P9 vs 1:*p*=0.0043, *t*=15.28). (**D**) The protein levels of ITGA9 in P3 MEFs and P9 MEFs. The quantitative results were shown on the right. Mean (±SEM), n=3, passed normality test: Shapiro-Wilk test; One sample *t* test (MEF P9 vs 1:*p*=0.0144, *t*=8.243). (**E**) The ITGA9 protein levels in MEF cells transfected with siNC and siMETTL3. The quantitative results were shown on the right. Mean (±SEM), n=3, passed normality test: Shapiro-Wilk test; One sample *t* test (siMETTL3 vs 1:*p*=0.034, *t*=5.282). (**F**) The ITGA9 protein levels in MEF cells infected with Lenti-NC and METTL3 lentivirus. The quantitative results were shown on the right. Mean (±SEM), n=3, passed normality test: Shapiro-Wilk test; One sample *t* test (METTL3 vs 1:*p*=0.0109, *t*=9.494). (**G**) The ITGA9 mRNA levels in MEF cells transfected with siNC and siMETTL3, and MEF cells infected with Lenti-NC and METTL3 lentivirus. Mean (±SEM), n=3, passed normality test: Shapiro-Wilk test, ns: no significant difference, One sample *t* test (siMETTL3 vs 1:*p*=0.7899, *t*=0.3039; METTL3 vs 1:*p*=0.5277, *t*=0.7578). (**H**) MeRIP-qPCR was used to detect the m6A abundances within ITGA9 mRNA in MEF cells transfected with siNC and siMETTL3. Mean (±SEM), n=3, passed normality test: Shapiro-Wilk test; One sample *t* test (siMETTL3 vs 1:*p*=0.0056, *t*=13.29). (**I**) Precursor and mature mRNA levels of ITGA9 in MEF cells transfected with siNC and siMETTL3. Mean (±SEM), n=3, passed normality test: Shapiro-Wilk test, ns: no significant difference, One sample *t* test (siMETTL3 vs 1:*p*=0.4213, *t*=1.004; METTL3 vs 1:*p*=0.5158, *t*=0.7827). (**J**) The subcellular localization of mature mRNA of ITGA9 in MEF cells transfected with siNC and siMETTL3 by nucleocytoplasmic separation experiment. Mean (±SEM), n=3, passed normality test: Shapiro-Wilk test, ns: no significant difference, Unpaired *t* test (siMETTL3 vs siNC, *p*=0.9393, *t*=0.08110). (K, L) The ITGA9 protein levels in MEFs transfected with siNC or siMETTL3 and then treated with the proteasome inhibitor MG132 (K) and the protein synthesis inhibitor CHX (cycloheximide) (L) at the indicated times. (M, N) The ITGA9 protein levels in MEF cells infected Lenti-NC or METTL3 under MG132 treatment (M) and CHX treatment (N) at the indicated times.

The MeRIP-seq data showed a stable m6A peak within the ITGA9-mRNA-3'UTR, which was significantly enhanced in senescent MEFs compared to young MEFs (Fig. 4A). MeRIP combined with qPCR verified that the m6A level in the ITGA9-mRNA-3'UTR was significantly elevated in senescent MEFs, which was consistent with the results of MeRIP-seq (Fig. 4B). Furthermore, ITGA9 expressions during the aging process were monitored by qPCR and western blot. Fascinatingly, in comparison to young MEFs, the ITGA9 protein level was evidently alleviated in senescent MEFs, while the mRNA level was up-regulated Fig. 4C and 4D).

To probe into the relationship between ITGA9 and METTL3 expression, we determined the protein and mRNA expression levels of ITGA9 in MEFs transfected with either siMETTL3 or a METTL3 overexpression lentivirus. The results showed that METTL3 silencing substantially elevated the ITGA9 protein level, while the ectopic expression of METTL3 decreased the ITGA9 protein level (Fig.4E and 4F), indicating a negative correlation between METTL3 and ITGA9 protein expression levels. Nonetheless, the ITGA9 mRNA levels showed no significant difference when altering the METTL3 expression (Fig. 4G). The above results suggested that m6A modification might be involved in regulating ITGA9 expression during senescence at the post-transcriptional or translational level, rather than at the transcriptional level.

Moreover, we sought to elucidate the potential mechanism by which METTL3 regulates the expression of ITGA9. Initially, MeRIP combined with qPCR assay unraveled a diminished m6A level in the ITGA9 transcript after knock-down of METTL3 (Fig. 4H). In addition, the result of qPCR exhibited that both the mature mRNA and precursor mRNA levels of ITGA9 remained unaltered in the absence of METTL3 (Fig. 4I), indicating that m6A modification might not impact on the transcription, splicing, or stability of ITGA9 mRNA. Nucleo-cytoplasmic separation assay revealed that METTL3 silencing showed no significant difference in the subcellular localization of ITGA9 mature mRNA (Fig. 4J), which suggested that METTL3 knock-down did not affect the transport of ITGA9 mRNA.

Then MEFs transfected with either siMETTL3 or siNC were treated with cycloheximide (CHX), a protein synthesis inhibitor. The result of western blot showed that CHX treatment significantly alleviated the up-regulation of ITGA9 induced by METTL3 deficiency (Fig. 4L). Additionally, the proteasome inhibitor MG132 was introduced to treat MEFs transfected with siMETTL3 or siNC. The results uncovered a more evident up-regulation of ITGA9 triggered by METTL3 silencing under MG132 treatment (Fig. 4K). MEFs were further infected with METTL3 overexpressed lentivirus and then treated with either CHX or MG132. Consistently, the data showed that CHX treatment attenuated the downregulation of ITGA9 caused by METTL3 overexpression, while MG132 treatment aggravated the changes in ITGA9 following ectopic expression of METTL3 (Fig. 4M and 4N). In general, the above results indicated that METTL3 might suppressed the translation of ITGA9 *via* m6A modification.

### METTL3 accelerated cellular senescence by modulating ITGA9

**Figure 5.**
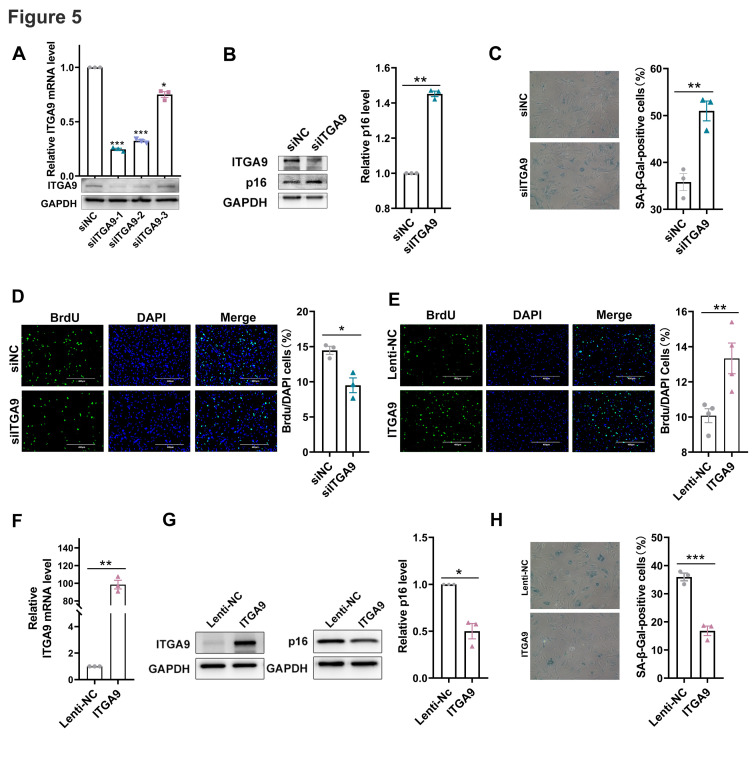
**ITGA9 staved off senescence in MEFs**. (**A**) The mRNA and protein levels of ITGA9 in MEF cells transfected with siNC and siITGA9-1/2/3.Mean (±SEM), n=3, passed normality test: Shapiro-Wilk test; One sample *t* test (siITGA9-1 vs 11: *p*=0.0002, *t*=68.12; siITGA9-2 vs 1: *p*=0.0003, *t*=63.15 ; siITGA9-3 vs 11: *p*=0.0111, *t*=9.410; ) (B) The p16 expression levels in MEF cells transfected with siNC and siITGA9. The quantitative results were shown on the right. Mean (±SEM), n=3, passed normality test: Shapiro-Wilk test; One sample *t* test (siITGA9 vs 1, *p*=0.0012, *t*=28.41). (**C**) Representative photographs of SA-β-gal staining of MEF cells transfected with siNC and siITGA9 (×100). The quantitative results of SA-β-gal staining were shown on the right. Mean (±SEM), n=3, passed normality test: Shapiro-Wilk test; Paired *t* test (siITGA9 vs siNC, *p*=0.0026, *t*=19.65). (**D**) Representative photographs of cells stained with DAPI (blue fluorescence) and BrdU (green fluorescence) in siNC and siITGA9 transfected MEF cells (scale bar: 400μm). The quantitative results of the ratio ( BrdU stained cells / DAPI stained cells) were shown on the right. Mean (±SEM), n=3, passed normality test: Shapiro-Wilk test; Paired *t* test (siITGA9 vs siNC, *p*=0.0144, *t*=4.138). (**E**) Representative photographs of cells stained with DAPI (blue fluorescence) and BrdU (green fluorescence) in Lenti-NC and ITGA9 infected MEF cells (scale bar: 400μm). The quantitative results of the ratio ( BrdU stained cells / DAPI stained cells) were shown on the right. Mean (±SEM), n=4, passed normality test: Shapiro-Wilk test; Paired *t* test (ITGA9 vs Lenti-NC, *p*=0.01, *t*=5.843). (**F**) The mRNA levels of ITGA9 in MEF cells infected with Lenti-NC or ITGA9 lentivirus. Mean (±SEM), n=3, passed normality test: Shapiro-Wilk test; One sample *t* test (ITGA9 vs 1, *p*=0.0025, *t*=20.08). (**G**) The ITGA9 and p16 protein expression levels in MEFs infected with Lenti-NC and ITGA9 lentivirus. The results of grayscale scanning were shown on the right. Mean (±SEM), n=3, passed normality test: Shapiro-Wilk test; One sample *t* test (ITGA9 vs 1, *p*=0.0256, *t*=6.124). (**H**) Representative photographs of SA-β-gal staining of MEF cells infected with Lenti-NC and ITGA9 lentivirus (×100). The quantitative results of SA-β-gal staining were shown on the right. Mean (±SEM), n=3, passed normality test: Shapiro-Wilk test; Paired *t* test (ITGA9 vs Lenti-NC, *p*=0.0009, *t*=33.30).

To explore the role of ITGA9 in the regulation of cellular senescence, siRNAs specifically targeting ITGA9 (designated as siITGA9-1/2/3) were designed and synthesized. The inhibition of ITGA9 in MEF cells was confirmed. Among these three siRNAs, siITGA9-1 displayed the highest suppression efficiency in MEFs, thus was employed in subsequent experiments, and designated as siITGA9 (Fig.5A). The results of western blot, SA-β-galactosidase staining and BrdU incorporation assay uncovered that the silencing of ITGA9 endowed MEF cells with enhanced senescence marker p16 levels and β-galactosidase activity, and reduced proliferative capacity (Fig.5B, 5C and 5D). These results indicated that ITGA9 deficiency significantly accelerated senescence in MEFs.

Subsequently, a lentivirus containing the coding sequence of ITGA9 was prepared (designated as ITGA9), and its overexpression of ITGA9 was confirmed by qPCR and western blot (Fig.5F and 5G). Expectedly, the ectopic expression of ITGA9 resulted in a significant reduction in p16 levels and β-galactosidase activity, but enhanced proliferative capacity of MEFs (Fig.5E, 5G and 5H). Overall, these results unveiled that ITGA9 staved off senescence in MEFs.

**Figure 6.**
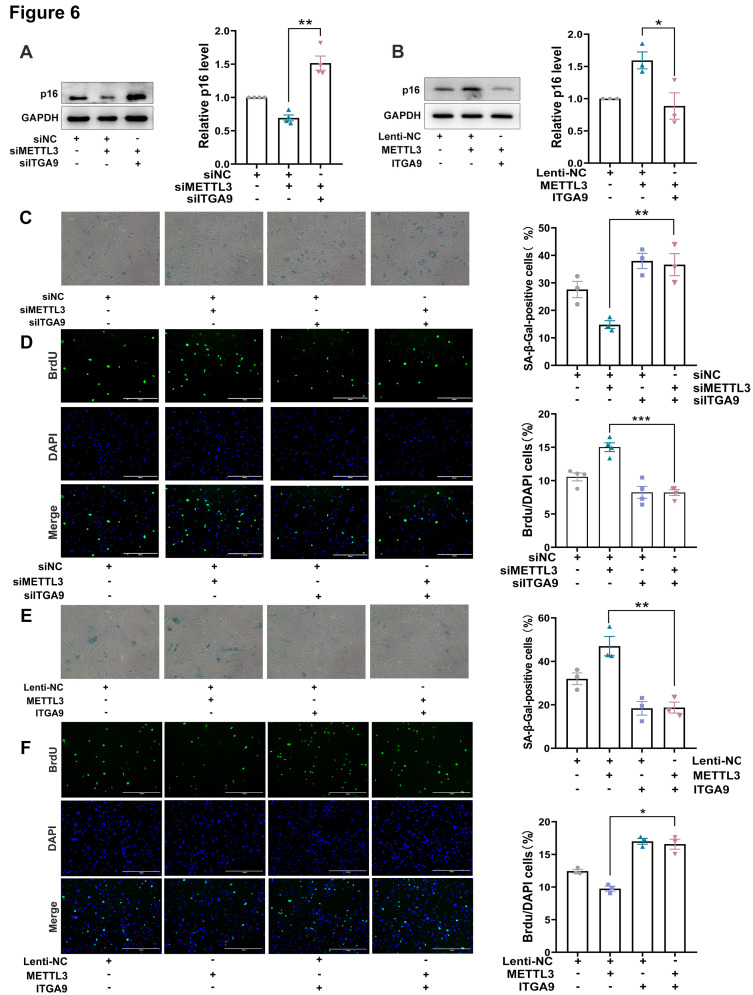
**METTL3 accelerated cellular senescence by modulating ITGA9**. (**A**) The p16 level in MEF cells transfected with siNC, siMETTL3 or siMETTL3 and siITGA9. The quantitative results were shown on the right. Mean (±SEM), n=4, passed normality test: Shapiro-Wilk test; Paired *t* test (*p*=0.0058, *t*=7.062). (**B**) The p16 level in MEF cells infected with Lenti-NC, METTL3 or METTL3 and ITGA9. The quantitative results were shown on the right. Mean (±SEM), n=3, passed normality test: Shapiro-Wilk test; Paired *t* test (*p*=0.017, *t*=7.563). (C, D) Representative photographs of SA-β-gal staining (C) and BrdU incorporation staining (D) of MEF cells transfected with siNC, siMETTL3, siITGA9, or siMETTL3 and siITGA9. The quantitative results were shown on the right. Mean (±SEM), passed normality test: Shapiro-Wilk test; Paired *t* test (C), n=3, *p*=0.0044, *t*=15.02; (D), n=4, *p*=0.0003, *t*=19.14). (E, F) Representative photographs of SA-β-gal staining (E) and BrdU incorporation staining (F) of MEF cells infected with Lenti-NC, METTL3, ITGA9 or METTL3 and ITGA9. The quantitative results were shown on the right. Mean (±SEM), n=3, passed normality test: Shapiro-Wilk test; Paired *t* test ((E): *p*=0.0095, *t*=10.18; (F): *p*=0.0231, *t*=6.469).

To illuminate whether METTL3 accelerated MEFs senescence *via* suppressing ITGA9, MEFs were co-transfected with siMETTL3 and siITGA9. The results revealed that siITGA9 dramatically reversed the effects of siMETTL3 on decreasing p16 levels and β-galactosidase activity, and enhancing cell proliferative capacity, suggesting that the inhibition of ITGA9 reversed the impact of METTL3 silencing on MEFs senescence (Fig. 6A, 6C and 6D). Moreover, co-infecting MEFs with ITGA9 and METTL3 overexpressed lentivirus showed that the overexpression of exogenous ITGA9 significantly counteracted the effects of ectopic METTL3 expression on p16 level, β-galactosidase activity and cellular proliferative capacity (Fig. 6B, 6E and 6F). In brief, these results concluded that METTL3 accelerated cellular senescence might be probably due to the negative regulation of ITGA9.

## DISCUSSION

With the in-depth exploration of the RNA field, accumulating evidence has revealed that post-transcriptional events of aging-related genes exert pivotal roles in the aging process. Non-coding RNAs, such as miRNAs [3], lncRNAs [4] and circRNAs [15] have been demonstrated to modulate the aging process through regulating aging-related genes at the post-transcriptional level. Recently, m6A methylation has been considered as an emerging post-transcriptional regulator of gene expression [5]. However, the roles and molecular mechanisms of m6A modification in cellular senescence remain poorly understood. Our results revealed that m6A level was dramatically up-regulated during the aging process, and METTL3 significantly accelerated cellular senescence. Furthermore, we unveiled that METTL3 exerted pivotal roles in cellular senescence might be probably due to the translational inhibition of ITGA9 in an m6A modification-dependent manner.

Recently, a growing body of evidence has indicated a potential relationship between m6A methylation and the progression of senescence. One group has reported that enhanced m6A modification in the FOS-mRNA-3’UTR triggered the up-regulation of FOS *via* suppressing the degradation of FOS-mRNA, which promoted the senescence of granulosa cells (GCs) and ultimately accelerating ovarian aging [16]. Another important work identified that METTL3-mediated m6A modification advanced the senescence of fibroblast-like synoviocytes (FLS) through decreasing the expression of autophagy-related 7 (AT7) by weakening AT7 mRNA stability [17]. On the contrary, m6A modification was downregulated in prematurely senescent human mesenchymal stem cells (hMSC), and this reduction in m6A level evidently decreased the MIS12 expression, which finally accelerated cellular senescence [18]. In addition, Kyung-Won et al. verified a decrease in overall m6A level in senescent peripheral blood mononuclear cells (PBMCs). And the knock-down of METTL3 or METTL14 contributed to the senescence of PBMCs [19].

The heterogeneity of METTL3 in the regulation of aging process and cellular senescence observed in the above studies might probably stem from cell-type specificity, microenvironment dependency (such as DNA damage stresses), the dynamic regulatory network of m6A modifications, dose effects and epigenetic crosstalk. Thus, the role of m6A modification in the aging process remains ambiguous and far from being elucidated. This study unveiled that m6A modifications were dramatically up-regulated during the aging process, and METTL3 promoted cellular senescence through suppression of ITGA9, which corroborated the critical impact of m6A modification in the aging process. Nevertheless, further investigations are still required to deepen the understanding of the central role and detailed mechanisms of m6A methylation in the regulation of aging.

**Figure 7.**
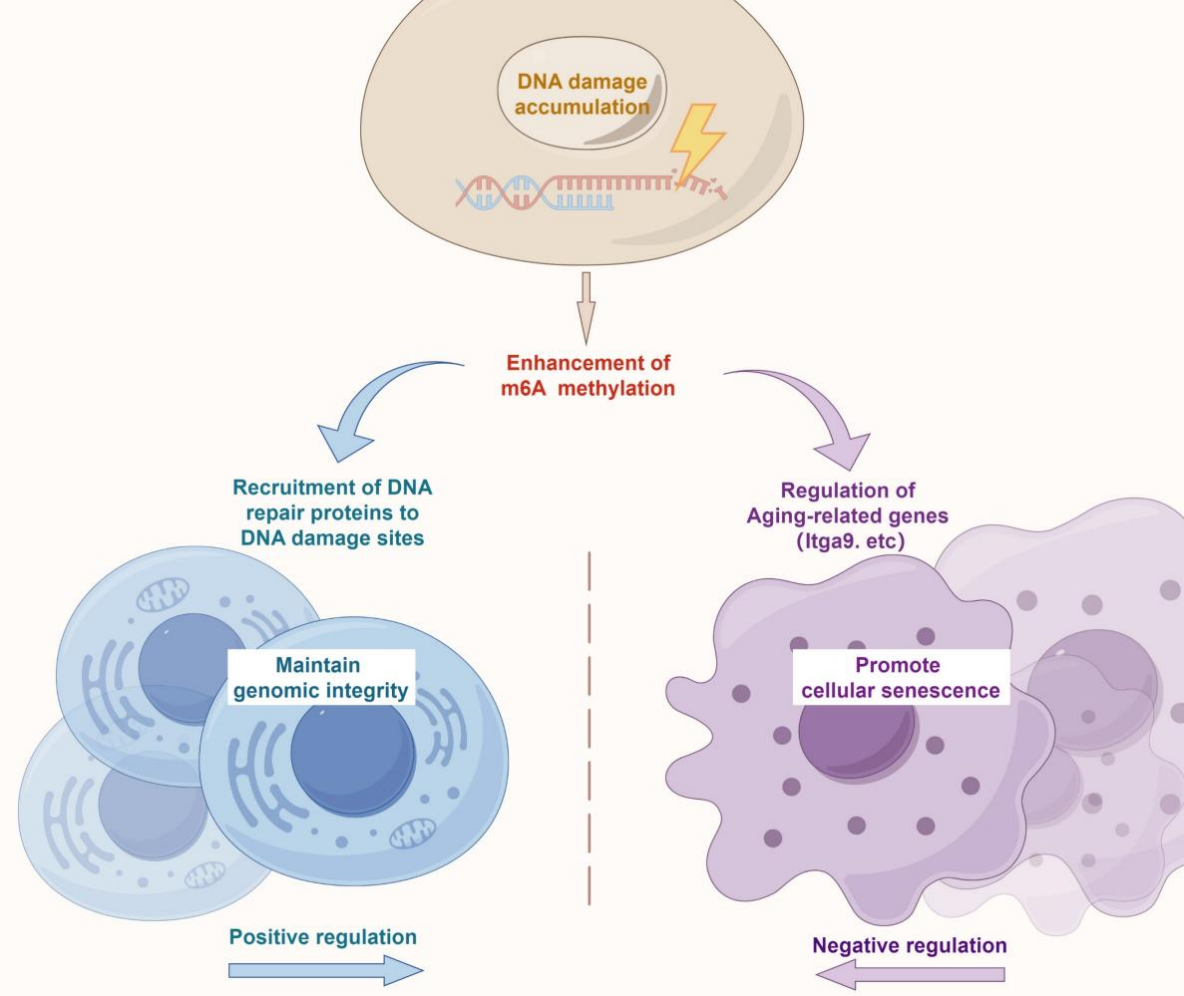
Schematic diagram of the dual role of m6A methylation in DNA damage repair and cellular senescence.

Previous literatures have established the close relationship between m6A modification and the DNA damage response. Xiang et al. has reported that m6A methylation occurred at single-stranded breaks (SSBs), where METTL3 recruited polκ to the damaged sites to facilitate effective repair of SSBs [7]. Yu et al. uncovered that H_2_O_2_-induced DNA damage lead to an increase in global mRNA m6A modification, which promoted the repair of DNA interstrand crosslinks and double-strand breaks (DSBs) [20]. Another group identified that METTL3 was phosphorylated by ATM and recruited to DSBs to catalyze the m6A modification of DNA damage-associated RNA, thus promoting homologous recombination-mediated DNA repair [21]. These studies suggested that m6A modifications might probably exert a protective effect against DNA damage. Alternatively, we herein presented evidence that the overall m6A methylation level was enhanced during the aging process, and ectopic expression of METTL3 significantly accelerated the cellular senescence. Consequently, we proposed a hypothesis that the accumulation of DNA damage accompanied by aging process caused a substantial increase in m6A modification levels, in order to maintain genomic integrity; but on the flip side of the coin, the enhanced m6A methylation modulated the expression of various aging-related genes and thus promoted cellular senescence (Fig 7). However, the threshold of DNA lesion accumulation, or whether there’re other key events to trigger the switch from DNA repair to an accelerated aging process was still far to be elucidated.

Multiple m6A binding proteins, referred to as “Readers”, have been identified, which specifically recognize and bind to m6A-modified mRNA, and then exert the regulatory functions to determine the fate of m6A-modified transcripts, including RNA splicing, nuclear transport, stability, and translation [22]. For instacnce, YTHDC1 regulated the splicing of target mRNAs by recruiting the pre-mRNA splicing factor SRSF3 (SRp20) and inhibiting the binding of SRSF10 (SRp38) to mRNA [23]. In addition, YTHDC1 mediated the nuclear export of bound mRNAs *via* incorporating target mRNAs into the nuclear mRNA export receptor NXF1 through interaction with SRSF3 [24]. YTHDF2 has been demonstrated to regulate mRNA stability in an m6A-dependent manner [25]. Eukaryotic initiation factor 3 (eIF3) has been identifed as a m6A reader protein, which initiated translation by recruiting 43S complex through binding with 5′UTR m6A [26]. Despite no m6A reader has been identified to mediate translational repression, m6A has been reported to inhibit translation by preventing m6A-modified mRNA from binding to ribosomes [27]. Another group has reported that 5'UTR-m6A in CTNNB1 suppressed translation efficiency [28]. The demonstration that m6A modification might negatively regulate ITGA9 through suppressing its translation, further verified the effect of m6A modification on translation suppression. Nevertheless, the identification of specifc m6A readers and the detailed mechanism required further investigation.

Integrin α9 subunit (ITGA9) belongs to the alpha integrin family and forms a heterodimeric transmembrane glycoprotein with the β1 subunit (α9β1), functioning as a receptor for VCAM-1 [29]. ITGA9 has been reported to be upregulated in various cancers, including glioblastoma, melanoma, hepatocellular carcinoma, and prostate cancer, and is closely associated with angiogenesis and lymph-angiogenesis [30], and cell proliferation and migration [31], suggesting a potential role of ITGA9 in tumor development [32]. Additionally, ITGA9 plays a critical role in mediating cell adhesion, an essential process in the inflammatory response. Recent studies have highlighted the significance of integrins in immune cell trafficking and their contribution to various inflammatory diseases [33]. For instance, the autocrine and paracrine interactions between α9β1 integrin and tenascin-C within the joint tissue microenvironment are pivotal in the pathogenesis of rheumatoid arthritis (RA) [34, 35]. Furthermore, the expression of ITGA9 has been shown to be regulated by various cytokines [36], suggesting a complex interplay between immune signaling and integrin expression. However, the potential role of ITGA9 in the aging process remains completely unknown. The present study uncovered that ITGA9 might probably be regulated by m6A modification *via* translation inhibition, and exerted critical role in modulating cellular senescence, which thereby greatly contributed to a more comprehensive understanding of ITGA9's function.

In this study, we have demonstrated that METTL3 accelerated cellular senescence by suppressing ITGA9 expression in an m6A modification-dependent manner. This work conferred a deeper understanding of the complex mechanisms of cellular senescence and provided novel insights and potential targets for delaying the aging process and preventing age-related diseases. Nevertheless, the m6A methylation sites within ITGA9 transcripts and the corresponding m6A reader proteins have yet to be identified. We would take that as a cut-off point for further exploring the detailed mechanism of m6A modification in regulating ITGA9 in the following study.

## Supplementary Materials



## Data Availability

The data supporting the findings of this study are available within the article, supplementary materials and public database. RNA sequencing data has been submitted to GEO database under accession number GSE281852.
