# Supplementary material for: The Flip Side of the Coin: METTL3 Serves as a Novel Cellular Senescence Accelerator via Negative Regulation of ITGA9
Source: Aging Dis. 2025 Mar 18;17(2):1034–51. doi: 10.14336/AD.2024.1715 (PMC12834426; doi:10.14336/AD.2024.1715)
Supplement: Supplementary file 1 [file AD-17-2-1034-s.pdf]

SUPPLEMENTARY DATA

The Flip Side of the Coin: METTL3 Serves as a Novel Cellular Senescence Accelerator via Negative Regulation of ITGA9

Yuting Li, Linying Huang, Miaochun Fang, Liwen Ye, Haiqing Yang, Weijia Wu, Yuan Yuan, Kun Cao, Hui-ling Zheng, Xuerong Sun, Yun Wu, Xing-Dong Xiong, Xinguang Liu, Shun Xu

© 2025. Li Y et al. Published online at http://www.aginganddisease.org/EN/10.14336/AD.2024.1715

SUPPLEMENTARY DATA

Supplementary Table 1. Primers for qRT-PCR

Name	Sense primer sequence (5'-3')	Antisense primer sequence (5'-3')
β-actin	AGATGACCCAGATCATGTTTGAG	AGAGCATAGCCCTCGTAGAT
METTL3	TTGTCTCCAACCTTCCGTAGT	CCAGATCAGAGAGGTGGTGTAG
ITGA9	AGGAATTGCCAATCTGAGGACTG	CCCTGGAGACATTAAAGGACACG
Pre-ITGA9	TCCAGTCCTAACGCAGAGTGT	CCCAGTCCCAACCATCTTCATT
Mature-ITGA9	AGGAATTGCCAATCTGAGGACTG	CCCTGGAGACATTAAAGGACACG

Supplementary Table 2. siRNA sequences

Name	siRNA sequences
siNC	UUCUCCGAACGUGUCACGU
siMETTL3-1	GGAGAUCCUAGAGCUAUUA
siMETTL3-2	CUGCACUUCAGACGAAUUA
siMETTL3-3	GCUACCGUAUGGGAACAUUA
siITGA9-1	CCUGUACGAAGAGUAUAA
siITGA9-2	GCAUUGAUAUGGAUGGAAA
siITGA9-3	GCAGGGCAGGUCUCAGAAATT

© 2025. Li Y et al. Published online at http://www.aginganddisease.org/EN/10.14336/AD.2024.1715

SUPPLEMENTARY DATA

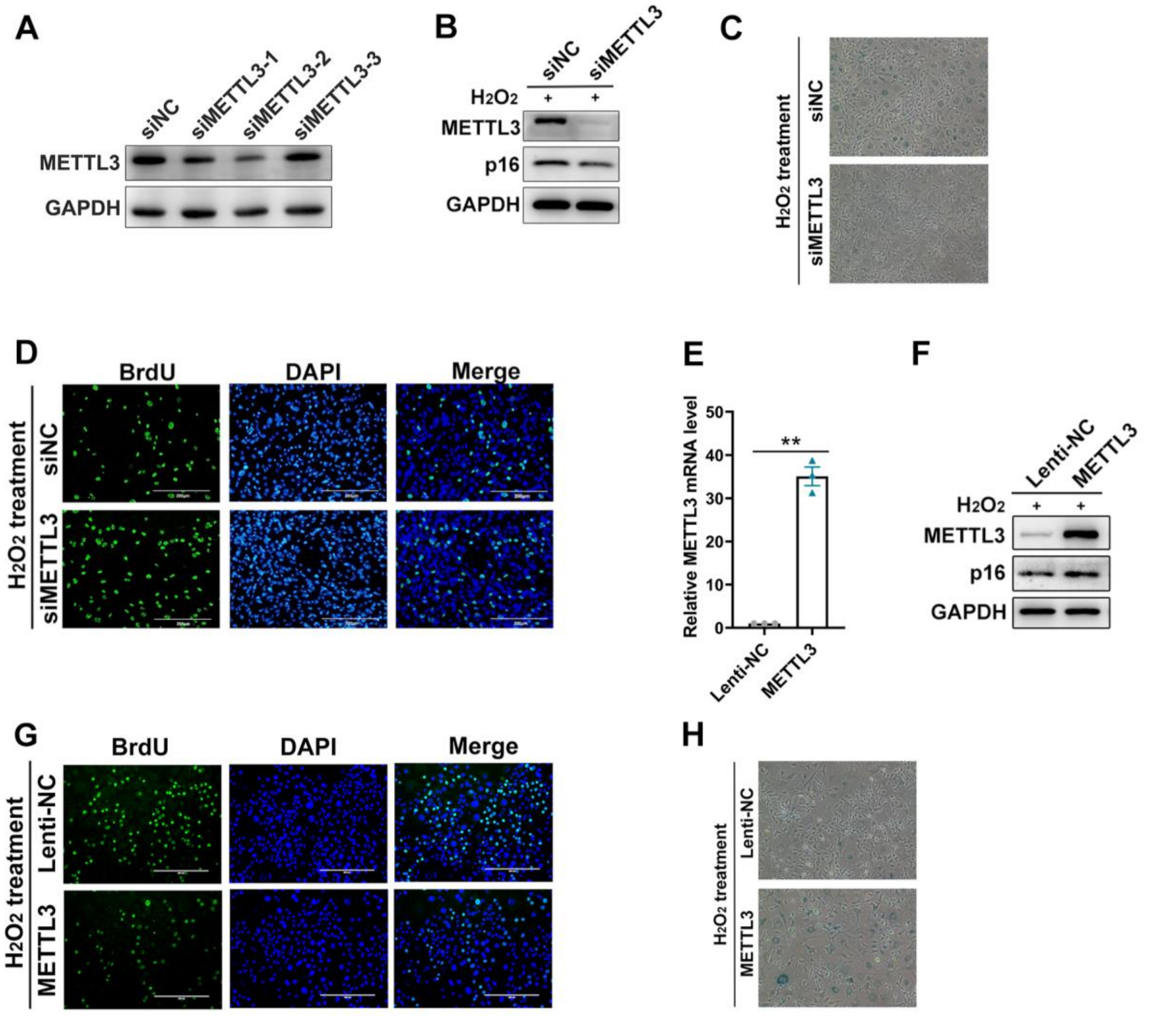

Supplementary Figure 1. METTL3 accelerated senescence induced by H_2O_2 in NIH/3T3 cells (A) Protein expression level of METTL3 in siNC or siMETTL3 transfected NIH/3T3 cells. **(B)** The p16 level in NIH/3T3 cells transfected with siMETTL3 under H_2O_2 treatment at indicated times. **(C)** Representative photographs of SA-β-gal staining of NIH/3T3 cells transfected with siNC and siMETTL3 under H_2O_2 treatment (×100). **(D)** Representative photographs of cells stained with DAPI (blue fluorescence) and BrdU (green fluorescence) in siNC and siMETTL3 transfected NIH/3T3 cells (scale bar: 200μm). **(E)** The METTL3 mRNA levels in NIH/3T3 cells infected with Lenti-NC or METTL3 lentivirus. Mean (±SEM), n=3, One sample *t* test (METTL3 vs 1, p=0.004, t=15.79) **(F)** The p16 and METTL3 protein levels in NIH/3T3 cells infected with Lenti-NC or METTL3 under H_2O_2 treatment. **(G, H)** Representative photographs of BrdU incorporation staining (G) and SA-β-gal staining (H) of NIH/3T3 cells infected with Lenti-NC, or METTL3.

© 2025. Li Y et al. Published online at http://www.aginganddisease.org/EN/10.14336/AD.2024.1715

SUPPLEMENTARY DATA

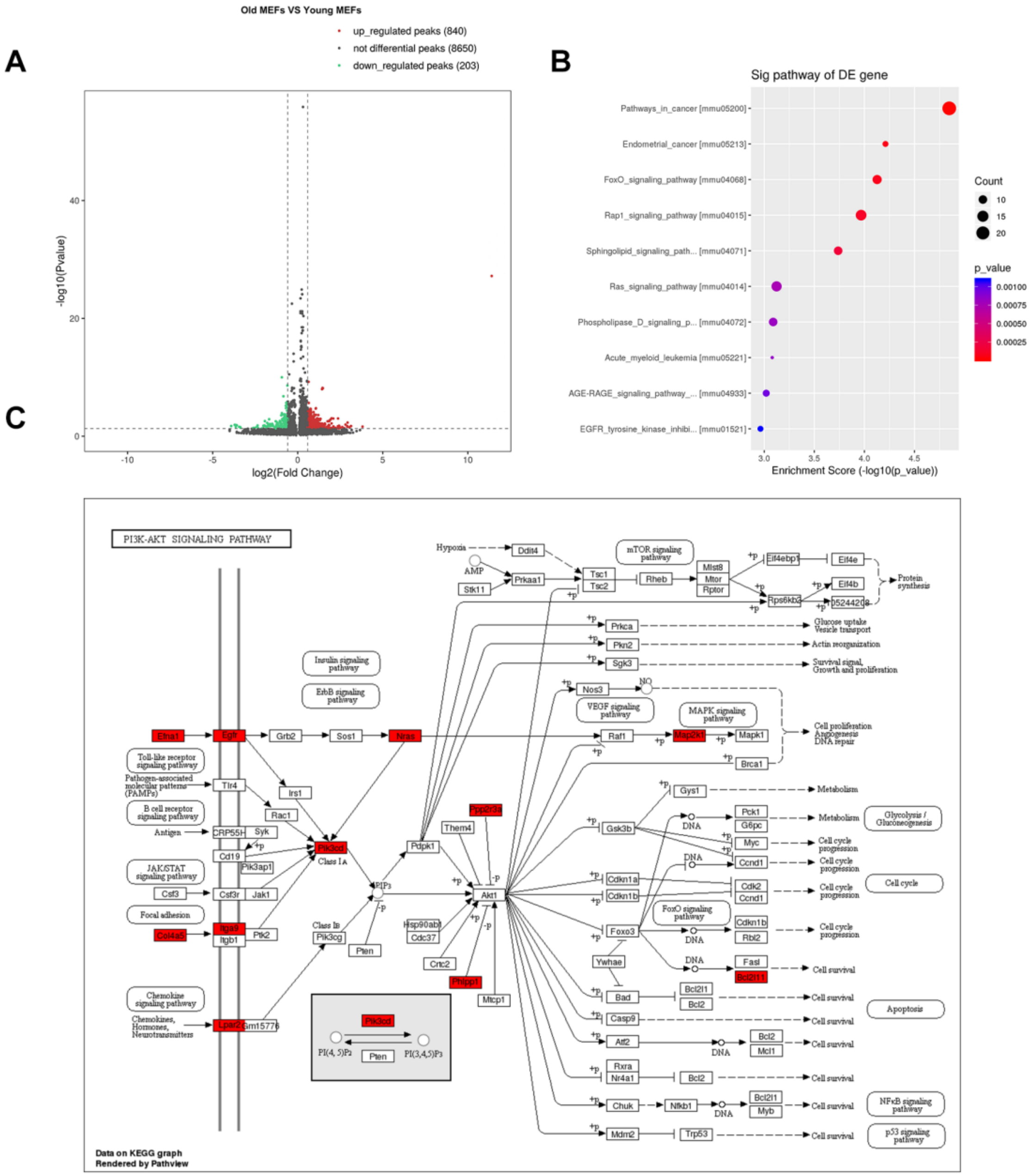

Supplementary Figure 2. Transcriptome-wide profiling of m6A modifications in MEFs replicative senescence (A) MeRIP-seq results showing volcano maps of differentially expressed genes in P3 MEFs and P9 MEFs (n=3). **(B)** KEGG pathway analyses of differentially m6A modified genes in in P3 MEFs and P9 MEFs. **(C)** Targets of m6A modification (highlighted in red) that enriched in the PI3K-AKT signaling pathways.

© 2025. Li Y et al. Published online at http://www.aginganddisease.org/EN/10.14336/AD.2024.1715
